# Gut Microbiota and Diabetic Complications: Potential Mechanisms, Microbial Signatures, and Clinical Implications

**DOI:** 10.3390/microorganisms14061285

**Published:** 2026-06-06

**Authors:** Christos G. Nikolaidis, Despoina Gyriki, Elisavet Stavropoulou, Eleni Karlafti, Triantafyllos Didangelos, Christina Tsigalou, Anastasia Thanopoulou

**Affiliations:** 1 Diabetes Center, 1st Propaedeutic Department of Internal Medicine, Medical School, ‘AHEPA’ University General Hospital, Aristotle University of Thessaloniki, 54124 Thessaloniki, Greece; linakarlafti@hotmail.com (E.K.); didang@auth.gr (T.D.); 2 2nd Department of Medicine, Hippokration Hospital, National and Kapodistrian University of Athens, 14564 Athens, Greece; a_thanopoulou@hotmail.com; 3 Hepatogastroenterology Unit, Academic Department of Internal Medicine, General Oncology Hospital of Kifissia “Agioi Anargyroi”, National and Kapodistrian University of Athens, 14564 Athens, Greece; 4 Master Program in “Food, Nutrition and Microbiome”, Laboratory of Hygiene and Environmental Protection, Department of Medicine, Democritus University of Thrace, 68100 Alexandroupolis, Greece; elisabeth.stavropoulou@gmail.com (E.S.); ctsigalo@med.duth.gr (C.T.); 5 Infectious Diseases Service, Department of Medicine, Lausanne University Hospital, University of Lausanne, 1015 Lausanne, Switzerland; 6 Laboratory of Hygiene and Environmental Protection, Department of Medicine, Democritus University of Thrace, Dragana, 68100 Alexandroupolis, Greece

**Keywords:** type 2 diabetes mellitus, gut microbiota, diabetic complications, chronic inflammation, intestinal permeability, inflammaging, short-chain fatty acids, TLR4 signaling

## Abstract

Type 2 diabetes mellitus is a systemic metabolic disorder with an extensive spectrum of complications, which still persist despite improvements in glycemic control. Emerging evidence suggests that gut dysbiosis may be an underpinning factor in the pathogenesis of both microvascular and macrovascular complications associated with diabetes. This narrative review explores the relationship between gut microbiota and the development of diabetes complications, including nephropathy, retinopathy, neuropathy, cardiovascular, cerebrovascular, peripheral vascular, and reproductive system disorders. First, existing evidence regarding the nature of shared and organ-specific microbial patterns is summarized. Next, key mechanistic pathways of inflammation and metabolism underlying tissue damage induced by dysbiosis are illustrated. Lastly, the role of gut microbiota and inflammaging as modifiers of these processes is described. Emerging clinical and translational implications are finally discussed, underscoring the promises of microbiota-based diagnostics as well as therapeutics that could serve as add-on approaches to the management of diabetic complications, alongside the application of artificial intelligence-based approaches to microbiome data analysis which may enhance biomarker discovery and risk stratification. Overall, although most evidence remains associative, increasing data support that gut microbiota dysbiosis may represent a potential disease modifier in the development of various diabetic complications. Further longitudinal and mechanistic studies are needed to clarify causality and to evaluate the clinical utility of microbiome-targeted interventions, including AI-assisted predictive models, in preventing or mitigating diabetic complications.

## 1. Introduction

Type 2 diabetes mellitus (T2DM) is a systemic metabolic disease that is characterized by hyperglycemia and multi-organ complications [1]. These complications contribute to increased morbidity and mortality [1,2]. Although there has been an overall improvement in glycemic outcomes, cases of microvascular and macrovascular complications of diabetes, such as those involving the kidneys, the eyes, nervous system, and cardiovascular and cerebrovascular systems, continue to be highly prevalent, implying that other pathways also play a significant role, in addition to those involving glucose control [2,3].

The gut microbiome represents an important modulator of host metabolic function, immune homeostasis, and barrier integrity, and it has been implicated in the pathobiology of diabetes and aging-related disorders through mechanisms involving insulin resistance, metabolic endotoxemia, and chronic low-grade inflammation [4]. Increasing evidence suggests that gut dysbiosis may contribute as the causative factor behind enhanced gut permeability, endotoxin leakage, or systemic inflammation that plays crucial roles in linking T2DM to its systemic complications [4].

Although each of these diabetic complications has been independently associated with gut microbiota alterations, the literature remains fragmented, with a single-organ approach and/or isolated mechanisms. There is a lack of complication-oriented synthesis of microbial signatures and shared mechanistic pathways [5,6]. The present review outlines an integrated overview of the involvement of gut microbiota in the pathogenesis of diabetic complications, summarizing common inflammatory and metabolic mechanisms, organ-specific microbial patterns, and emerging clinical implications.

## 2. Materials and Methods

This is a narrative literature review integrating evidence from selected studies identified through systematic searches of PubMed, Scopus, and Google Scholar, covering articles published up to March 2026. The following search terms were used alone or in combination for the literature review: “type 2 diabetes mellitus,” “diabetic complications,” “gut microbiota,” “gut dysbiosis,” “intestinal permeability,” “metabolic endotoxemia,” “lipopolysaccharide,” “TLR4 signaling,” “chronic inflammation,” “inflammaging,” “immunosenescence,” “short-chain fatty acids,” “bile acids,” “trimethylamine N-oxide,” “cardiovascular disease,” “diabetic nephropathy,” “diabetic neuropathy,” “diabetic retinopathy,” “cerebrovascular disease,” “animal models,” “in vitro studies,” and “clinical studies.” Boolean operators (AND, OR) were used to refine and combine search terms. Articles were selected based on their relevance to the role of gut microbiota–host interactions in the pathogenesis of microvascular and macrovascular complications in type 2 diabetes. Exclusion criteria included non-English publications, case reports, conference abstracts, commentaries, and editorials.

## 3. Conceptual Framework: From Dysbiosis to Organ Damage

Dysbiosis of the gut microbiota in T2DM is characterized by reduced microbial diversity, changes in certain microbial populations, reduced production of beneficial metabolites such as short-chain fatty acids (SCFAs), loss of barrier function, and enhanced levels of inflammation [4]. Moreover, the alterations in gut microbiota associated with aging further modulate the immune and inflammatory responses of the host and further increase the risk of chronic complications [7].

## 4. Gut Microbiota-Associated Diabetic Complications

**Figure 1:**
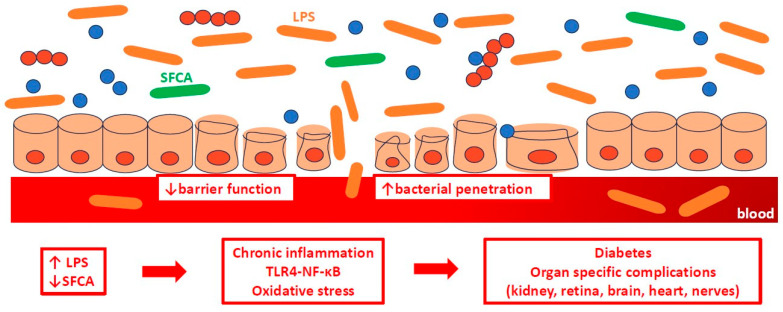
Gut dysbiosis increases intestinal permeability and shifts the microbiota toward LPS-producing Gram-negative bacteria (orange) while reducing SCFA-producing bacteria (green). Circulating LPS activates the TLR4-NF-κB pathway, leading to pro-inflammatory cytokine production sustaining chronic low-grade inflammation. Concomitant SCFA depletion further compromises epithelial barrier integrity and amplifies inflammatory signaling, while indirectly modulating oxidative stress. Together, these processes impair insulin signaling, promote β-cell dysfunction, and contribute to the onset and progression of diabetes and its complications. Horizontal arrows indicate the proposed pathogenic sequence linking gut dysbiosis to diabetes and its complications. Upward arrows denote increased levels or activity, whereas downward arrows denote decreased levels or activity of the indicated factors.

Restoring gut microbiota balance through controlled management of diabetes can efficiently reduce the risk of severe diabetic complications. Gut microbiota dysbiosis has been associated with complications such as diabetic retinopathy, nephropathy, neuropathy, peripheral vascular disease, cardiovascular disease, and cerebrovascular disease (Figure 1). Each condition is linked to bacterial dysbiosis, increased inflammation, oxidative stress, and other metabolic disturbances. Gut microbiota eubiosis prevents or delays such complications. Balance, diversity, and stability of gut microbiota are prime factors in improved metabolic health, reduction of inflammation, and enhancement of vascular function.

The gut microbiome has been implicated in a range of diabetes microvascular complications, including diabetic nephropathy, diabetes-associated cognitive decline, diabetic retinopathy, and diabetic peripheral neuropathy [6]. It also appears to contribute to macrovascular disease in diabetes as well as diabetes-related reproductive system impairments [8,9]. Taken together, the evidence demonstrates that gut microbiota and microbial metabolites have a significant role in diabetes complications [10].

Below is a summary of the existing information connecting gut microbiota to specific diabetic complications; nevertheless, most of the studies conducted so far have only shown associative connections.

### 4.1. Diabetic Nephropathy

Diabetic nephropathy (DN) is a multi-factorial complication of T2DM that typically progresses to end-stage renal disease (ESRD) in spite of aggressive control of glycemia, blood pressure, and other risk factors [11]. Treatments targeting oxidative stress and inflammation through multiple pathways with agents like vitamin D receptor activators (VDRA) and incretin-related molecules and valid biomarkers are urgently needed for measuring progression [11].

There is significant evidence that connects gut microbiota to diabetes, obesity, and kidney disease, with new research suggesting a link between dysbiosis and diabetic kidney disease (DKD) [12,13]. While the exact causal relationship between gut dysbiosis and DKD is still unclear, natural interventions (like dietary changes, prebiotics, probiotics, and fecal microbiota transplantation) present a promising, safe, and accessible way to enhance glycemic control and prevent complications [14]. Dysbiosis is linked to endotoxemia and chronic inflammation, mainly due to impaired intestinal barrier function and a reduction in beneficial SCFA-producing bacteria [15,16] as well as an increase in Gram-negative, lipopolysaccharide (LPS)-producing bacteria leading to translocation into the systemic circulation, thus triggering systemic inflammation [17]. Both T2DM and CKD/ESRD, which frequently occur together in the clinical setting, share similar detrimental effects on the intestinal microbiota composition and function [18]. Dysbiosis in nephropathy promotes uremic toxin generation (indoxyl/p-cresyl sulfates) and impairs the gut barrier, exacerbating kidney injury [19].

DKD patients present with an increased abundance of Proteobacteria, which may promote disease progression by modulating LPS production, SCFA production, and carbohydrate metabolism, processes that can trigger chronic low-grade inflammation through macrophage, monocyte, and neutrophil activation [20].

Zhang et al.’s study [21], using metagenomic sequencing, indicates that gut microbiota is involved in diabetic nephropathy pathogenesis: both T2DM and DN patients suffer from severe reductions of butyrate-producing bacteria (*Roseburia intestinalis*, *Clostridium*, and *Eubacterium*), potential probiotics (*Lachnospira* and *Intestinibacter*), and some microbial taxa that are strongly correlated with clinical markers such as BMI, HbA1c, and LDL-C. In addition, some gut microorganisms not only act as diagnostic indicators for DN (*Clostridium* sp. CAG_768, *B. propionicifaciens*, and *Clostridium* sp. CAG_715) but also modulate major metabolic pathways that underlie the base of the disease’s pathophysiology—the citrate cycle, bile acid biosynthesis, and amino acid metabolism.

Gut microbiota is a key indicator for diabetic nephropathy development and progression in the initial stage [22]. Specifically, *Alistipes*, *Bacteroides*, *Subdoligranulum*, *Lachnoclostridium*, and the *Ruminococcus torques* group are considered to be harmful factors that can exacerbate the progression and development of DN [23]. Patients with diabetic nephropathy demonstrated significant dysbiosis with high reduction in gut bacterial richness and diversity from phylum to genus level [24]. Fecal microbiome analysis also revealed distinctive microbial signatures differentiating healthy individuals from DN patients (stage 3 and stage 4) with gender- and BMI-correlated variations, with *Megasphaera*, *Veillonella*, *Escherichia-Shigella*, *Anaerostipes*, and *Haemophilus* showing as potential new biomarkers for the severity of DN [25].

Finally, changes in gut microbiota have been recognized as accountable for DN occurrence in patients with biopsy-proven DN [26]. Specifically, *Prevotella* concentration in fecal samples discriminates DM subjects from controls, while *Escherichia-Shigella* and *Prevotella* fluctuations distinguish DN from DM and can help in the physiopathological diagnosis of DN [27].

### 4.2. Diabetic Retinopathy

Diabetic retinopathy (DR) is among the serious complications of DM and is the leading cause of blindness in individuals of working age, while the current treatment modalities address only late microvascular damage [28]. The most recent evidence blames gut dysbiosis with the onset of low-grade inflammation on the gut–retina axis as a suspected etiology for the onset of DR [28]. The review by Alarcón Yempén et al. outlines the biochemical mechanisms involved in DR and suggests that modulation of the gut–retina axis is a promising new therapeutic and prevention strategy [29].

In a cross-sectional study of 612 patients with T2DM, the prevalence of malnutrition was between 10.0% and 34.3% in total and between 16.3% and 45.1% in DR patients based on various nutritional assessment tools (Global Leadership Initiative on Malnutrition (GLIM) criteria, controlling nutritional status (CONUT), nutritional risk index (NRI), and prognostic nutritional index (PNI)). Malnourished individuals had profoundly raised chances of both the presence and severity of DR (with adjusted odds ratios ranging from 1.67 to 2.24), highlighting the importance of nutritional evaluation as well as prompt treatment in order to prevent DR development or onset [30].

A comprehensive metabolomics study has identified that changes in the concentration of metabolites (such as elevated 12-hydroxyeicosatetraenoic acid and 2-piperidone and reduced antioxidants such as carnosine, succinate, nicotinic acid, and niacinamide) are linked with the onset and progression of DR [31]. Of particular interest, gut microbiota control the generation of these metabolites, and their dysregulation may trigger oxidative stress and inflammation, while disturbances in bile acid metabolism (evidenced by changes in taurochenodeoxycholate and tauroursodeoxycholate) also play a role in DR pathology, and therapies like intermittent fasting and tauroursodeoxycholate treatment have therapeutic implications [8].

Zhou et al. compared fecal samples among DR patients, T2DM patients without retinopathy, and healthy controls and observed that gut microbiota composition was significantly different. DR patients presented with increased abundance of *Faecalibacterium*, *Roseburia*, *Lachnospira*, and *Romboutsia* genera and decreased levels of *Akkermansia*, with typical fecal metabolite profiles [32].

The gut microbiota in DR patients exhibited significant differences from healthy controls, with reduced microbial diversity in DR patients [33]. These changes included imbalances of the Firmicutes, *Bacteroidetes*, *Synergistota*, and *Desulfobacterota* phyla and increased levels of *Bacteroides*, *Megamonas*, *Ruminococcus_torques_group*, *Lachnoclostridium*, and *Alistipes*, with reduced levels of *Blautia*, *Eubacterium_hallii_group*, *Collinsella*, *Dorea*, *Romboutsia*, *Anaerostipes*, and *Fusicatenibacter* [34].

In a study by Huang et al., clinical data and fecal samples were collected from 75 participants, including diabetic patients both with and without retinopathy, as well as healthy controls [33]. The gut microbiota was characterized using 16S rRNA gene sequencing. The findings showed that both groups of diabetic patients had lower alpha and beta diversity compared to the healthy controls, along with significant changes in microbial composition [33]. Specifically, there were increased levels of *Bifidobacterium* and *Lactobacillus*, while genera such as *Escherichia-Shigella* and *Faecalibacterium* were found to be less abundant. Additionally, a set of 25 bacterial family biomarkers was identified to differentiate the retinopathy group, with *Pasteurellaceae* standing out as a key individual predictor (AUC 0.74). Moreover, 14 family biomarkers were significantly linked to fasting blood glucose levels, mostly showing a negative correlation [33]. In DR patients, both gut bacterial richness and diversity were severely depleted when compared to diabetic non-retinopathic patients and healthy controls, with DR patients showing overrepresentation of taxa such as *Pseudomonas*, *Ruminococcaceae*-UCG-002/005 and *Christensenellaceae*-R-7 and increased microbial pathways for glucose and nicotinate degradation. Parallel untargeted fecal metabolomics identified extensive accumulations of amino acids (arginine, ornithine, and serine) and arachidonic acid in the DR patients—processes associated with heightened arginine and lysine catabolism—and showed strong positive correlations between *Ruminococcaceae*-UCG-002/*Christensenellaceae*-R-7 abundance and fecal L-arginine and ornithine levels [35].

Lastly, findings establish a causative relationship between DR and specific gut microbiota taxa, namely *Christensenellaceae*, *Peptococcaceae*, *Ruminococcaceae*_UCG_011, *Eubacterium_rectale*_group, and *Adlercreutzia*. Such microbial strains have potential as new biomarkers and are informative in the prevention and treatment of DR [36].

### 4.3. Diabetic Neuropathy

Neuropathic pain (NP) is a long-term illness caused by damage or disease affecting the somatosensory nervous system, leading to long-term physical and psychological pain [37]. Growing evidence in recent years has highlighted the significant role of gut microbiota in diseases, such as NP. Gut microbiota influences immune, neural, endocrine, and metabolic pathways and forms a network that can influence NP development either directly or indirectly [38]. This information opens up new therapeutic options for interventions, such as drugs and dietary modification, that aim to modulate pain through the gut microbiota [39].

Emerging evidence presents a complex, two-way interaction between gut microbiota and neuropathic pain mediated by the vagus nerve, immune mechanisms, and other metabolites. Preclinical evidence indicates that therapeutic interventions like probiotics, fecal microbiota transplantation (FMT), diet change, and certain supplements could prevent neuropathic pain in a variety of conditions [40,41,42]. But the field is still in its infancy since high model heterogeneity and limited clinical translations mar the field. Future research with standardized procedures and interdisciplinary cooperation is needed to establish causal relationships and evidence-based, clinically useful treatments [42].

At the phylum level, diabetic neuropathy patients exhibit increased abundance of Firmicutes and Actinobacteria and decreased abundance of *Bacteroidetes* [43]. At the genus level, there is a stark decrease in beneficial bacteria such as *Bacteroides* and *Faecalibacterium* with increased abundance of potential pathogens such as *Escherichia-Shigella*, *Lachnoclostridium*, *Blautia*, *Megasphaera*, and the *Ruminococcus* torques group [43]. Notably, *Megasphaera* richness is positively linked with insulin resistance, while some bile acids (glycine ursodeoxycholic acid and tauroursodeoxycholic acid) are linked with the *Ruminococcus* gnavus group, *Phascolarctobacterium*, and *Parabacteroides*, reflecting a mechanistic link with insulin resistance and dyslipidemia [43]. In summary, type 2 diabetic patients with peripheral neuropathy have a significantly different intestinal microbiota compared to normal subjects, characterized by an imbalance towards conditional pathogens that may have a significant role in the etiology and development of neuropathy [43]. Findings by Huang et al. indicate that gut microbial dysbiosis characterized by chronically increased abundance of phyla *Mycobacterium*, *Turicibacter*, and Actinobacteria could be implicated in the pathogenesis of diabetic peripheral neuropathy with cognitive impairment [44].

A study by Du et al. found that T2DM-gastrointestinal autonomic neuropathy (GAN) patients (trending with increasing age, lowering triglycerides, and lowering BMI) had distinct gut microbiota profiles compared to healthy individuals as well as T2DM patients [45]. Healthy controls shared *Bacteroidetes*, Firmicutes, and Proteobacteria as the dominant phyla, while T2DM patients shared enrichment of *Fusobacteria*-related taxa, which have a role in carotenoid and flavonoid biosynthesis [45]. In contrast, T2DM_GAN patients were overrepresented by Gammaproteobacteria and Gammaproteobacterium-associated groups (e.g., *Enterobacteriales* and *Escherichia-Shigella*), which may assist in facilitating bacterial invasion and pathogenic E. coli infection, suggesting that GAN exacerbates gut dysbiosis in T2DM [45].

Advances in biomarkers and diagnostic tests have even more clearly delineated painful diabetic neuropathy, yet treatment remains more or less the same—glycemic control is the only therapy for diabetic neuropathy, and symptomatic treatment forms the cornerstone of treatment for painful diabetic neuropathy [46]. Follow-up of disease course and establishment of diagnostic tests require large prospective cohort studies, but precision medicine-informed pharmacologic studies can spawn novel disease-modifying therapies [47].

Overall, a review by Pane et al. identified a high level of association between gut microbiota changes and neuropathic disorders manifested as chronic pain, neurological impairment, and increased inflammatory mediator secretion [48]. Of note, results from seven studies indicated that normalization of microbiota homeostasis (e.g., with FMT) recovered a normal phenotype in animal models, suggesting that modulation of the gut microbiota may represent a promising strategy for the treatment of neuropathic and CNS diseases, which is worthy of further clinical investigation [48].

### 4.4. Cerebrovascular Disease

Diabetes mellitus is closely linked to atherosclerosis through shared processes like inflammation, oxidative stress, and advanced glycation end products (AGEs)-induced endothelial damage [49]. The combination significantly raises the occurrence of cardio- and cerebrovascular complications (especially stroke) in diabetic patients, among whom poorer outcomes and reduced benefit from standard therapy are more likely [50]. Experimental models illustrate that chronic hyperglycemia impairs cerebrovascular structure and function, highlighting therapeutic targets and the need for further studies to define these complex interactions [50].

Genetic predisposition to T2DM, as indicated by high HbA1c levels, is also associated with an increased risk of ischemic stroke (large artery and small vessel subtypes) and with markers of brain structural change such as carotid plaque, white matter damage, and atrophy [51]. Genetic pathways involved in insulin resistance are also associated with large artery and small vessel strokes [51]. Meanwhile, susceptibility to β-cell dysfunction is linked with small vessel stroke, intracerebral hemorrhage, and reduced gray matter and total brain volumes [51]. Dysbiosis caused by environmental and host factors disrupts the bidirectional interaction of the microbiota–gut–brain axis that leads to immune, metabolic, and nervous system dysfunctions that accompany age-related conditions and stroke susceptibility [52]. The connection between gut microbiota and the brain has led to significant advancements in neuroscience research. However, the vast gut microbiome requires further study to understand the role of gut microorganisms and their communities in neurodegeneration and neuroprotection [53].

The gut microbiota has emerged as a novel risk factor in cardiovascular and cerebrovascular disease [54]. Host microbial colonization, diet, and attendant metabolic changes modulate the growth of atherosclerotic plaques [55]. Additionally, engagement of innate immune signaling pathways (e.g., TLR2) is the trigger for platelet deposition and arterial thrombosis. Notably, the gut microbiota-derived metabolite trimethylamine N-oxide (TMAO) promotes plaque formation and prothrombotic platelet function, sustaining the connection of gut microbial content to cardiovascular risk [54].

Findings indicate that a genetically elevated abundance of Porphyromonadaceae is associated with reduced intracranial aneurysm (IA) risk [56]. Nominal causal associations were observed between different gut microbiota and IA and between several stroke subtypes [57]. Reverse Mendelian randomization analysis did not show that there was any significant causal connection between gut microbiota and IA, but it showed that any stroke and ischemic stroke with a genetically predicted connection were linked to lower abundances of *Clostridiaceae1* and Clostridiales [56]. Also, the genus *Streptococcus’s* influence on certain stroke subtypes is suspected to be mediated by T2DM, while the association between the genus *Eubacterium brachy* group and ischemic stroke is mediated by systolic blood pressure, with no heterogeneity or horizontal pleiotropy observed [56].

### 4.5. Coronary Heart Disease

Understanding the interaction of gut microbiota with other factors in the body is necessary to design proper interventions against hypercholesterolemia and coronary artery disease (CAD) [55,58,59]. Advanced technologies and comprehensive studies, including ethnicity and gender, are the most crucial tools for revealing bacterial mechanisms, resulting in effective, targeted microbiome-based therapies that could reduce the economic and social burden of CAD [60].

While the human gut microbiota generally keeps detrimental pathogens at bay, disturbances in its homeostasis have been linked to the development of cardiovascular diseases [61]. Metabolites derived from the gut, like SCFA, TMAO, bile acids, and polyphenols, are critical for cardiovascular wellness, and interventions aimed at them through diet, prebiotics, probiotics, or particular inhibitors offer an attractive avenue to prevent and treat CVD, especially among high-risk patients [62].

In a case–control study, CAD patients with advanced disease had reduced gut microbiome diversity compared to age- and demographic-matched controls [63]. Notably, reduced butyrate-producing taxa (*Lachnospiraceae* NK4B4 group and *Ruminococcus gauvreauii*) and the increase in *Ruminococcus gnavus* were associated with CAD even after adjusting for coronary risk factors such as dyslipidemia and diabetes. These alterations suggest that some changes in gut microbiota may contribute to the etiology of coronary artery disease [63].

A systematic review and meta-analysis showed that CAD patients exhibit profoundly reduced gut microbiome diversity, lower *Bacteroidetes* and *Lachnospiraceae* abundance, and higher Enterobacteriaceae, *Lactobacillus*, and *Streptococcus* abundance [64]. These microbiota alterations are linked with various bacterial metabolites that promote atherosclerosis and thereby increase the risk of CAD development and progression [64].

In a comparative study of congestive heart failure (CHF) advanced patients and controls, researchers observed extensive dysbiosis in gut microbiota [65]. Specifically, there was an extensive decrease in SCFA-producing bacteria like *Ruminococcaceae UCG-004*, *UCG-002*, the *Lachnospiraceae FCS020 group*, and *Dialister,* whereas there was an excessive abundance of *Enterococcus* species, which are high lactic acid producers [65]. Microbiome changes were correlated with a number of important functional pathways, such as cell cycle regulation, cell division, and SCFA-mediated metabolism [65]. Collectively, these data provide some insight into the pathogenesis of severe CHF and indicate that modulating gut microbiota composition may be an attractive therapeutic target [65].

Evidence shows that myocardial ischemia/reperfusion (I/R) causes gut dysbiosis and bacterial translocation, thereby perpetuating enhanced local and systemic inflammation, exacerbating I/R injury [66]. Pre-injury depletion of microbiota corroborates the contribution of translocated bacteria in triggering these inflammatory reactions. GLP-2 therapy of the heart–intestine axis also shows promise to reduce myocardial I/R injury [66].

### 4.6. Peripheral Vascular Disease

Peripheral arterial disease (PAD) is defined by partial or complete obstruction of peripheral arteries, typically in association with systemic atherosclerosis, and risk factors such as diabetes mellitus [67]. The risk of PAD is more than twofold higher in diabetic patients; its associated complications can lead to increased hospitalization rates, impaired quality of life, morbidity, and mortality [68]. Despite its clinical significance, PAD is vastly underdiagnosed and undertreated and consequently has spurred recent advances in its pathophysiology, epidemiology, diagnosis, and treatment [69].

Shi et al. used bidirectional Mendelian randomization analysis to reveal a causal link between gut microbiota changes and PAD, identifying risk factors as *Family XI*, *Lachnoclostridium*, and *Lachnospiraceae* UCG001 and protective factors as Actinobacteria, Acidaminococcaceae, and certain Ruminococcaceae groups [70]. The function of the intestinal barrier is disrupted in dysbiosis, making lipopolysaccharides capable of initiating TLR4-mediated inflammation in order to strengthen atherosclerosis [71]. In addition, microbial metabolites play central functions, with choline being bacterially metabolized by *Lachnoclostridium* into TMAO, which is a confirmed atherosclerotic risk factor. Ruminococcaceae, a family of SCFA-producing bacteria, generates butyrate, with anti-inflammatory and cholesterol-reducing effects. This emerging evidence is also shedding new light on targeted therapies for PAD [70].

Taken together, existing data indicate a tight correlation between diabetic PAD and gut microbiota [72,73]. Recent genetic analysis suggests that specific bacterial metabolic pathways (e.g., KETOGLUCONMET-PWY) may reduce PAD risk, with immune cells like regulatory T cells and B cells partially mediating these effects [74]. Modulating the microbiota through individualized antibiotic therapies, in addition to prebiotics, probiotics, and dietary measures, could widen the horizon for individualized treatment strategies of diabetic PAD [73].

### 4.7. Gut Microbiota, Diabetes, and the Reproductive System

The term “gut–reproductive axis” has appeared in the context of the two-way interaction between gut microbiota and reproductive health [75]. The gut microbiota has also appeared as a major regulator on the crossroads between metabolic health and reproductive health [76]. Studies have revealed that although overall gut microbiota is similar in individuals with and without erectile dysfunction, those with erectile dysfunction have higher levels of *Clostridium XVIII* and lower levels of *Alistipes* [77]. Endothelial activation markers such as E-selectin and a profile of inflammatory cytokines that reflect a higher TNF-α:IL-10 ratio have been found to be increased in clinical trials conducted among diabetic patients suffering from erectile dysfunction [78]. In diabetic models, the depletion of beneficial bacteria and elevation of inflammatory markers further suggest that gut imbalances can predispose to vascular inflammation and endothelial damage, leading to erectile dysfunction [79].

Overall, gut microbiota modification in a high-fat diet in mice was linked with reproductive damage in men [80]. A high-fat diet induces disorder in glucose metabolism and subsequently leads to reproductive dysfunction in mice [81]. Dysbiosis inhibits spermatogenesis and sperm motility, with mechanistic pathways related to increased conditions of endotoxemia, epididymal inflammation, and testicular gene expression alterations [81]. Catalpol has the ability to function as a drug with an incredible increase to gut microbiology to oppose reproductive damage resulting from the inhibition of glycolysis [82]. Also, fecal microbiota transplantations of microbiota communities derived from high-fat diet-fed mice to normal-diet-fed mice help to confirm these conditions [81]. Research is ongoing in this context for using microbiota-directed treatments to enhance diabetes and reproductive disease outcomes [76].

The complications span across microvascular (e.g., nephropathy, retinopathy, and neuropathy) and macrovascular (e.g., cardiovascular, cerebrovascular, and peripheral vascular diseases) types. Table 1 further adds reproductive system dysfunctions, an often-overlooked complication of metabolic and microbial imbalance. Every disease is associated with typical microbial alterations—e.g., reduction of SCFA-producing bacteria, increase in endotoxin-producing Proteobacteria, or altered microbial metabolites like TMAO—that contribute to inflammation, oxidative stress, and organ dysfunction. This emphasizes the key position of the gut microbiota not only in the etiology of diabetes but also in the severity and pathogenesis of its systemic complications.

### 4.8. Gut Microbiota, Aging, and Inflammaging in Diabetic Complications

Aging is one of the most important modifying factors in the development of type 2 diabetes mellitus and its related complications and is closely linked with gut microbiota, which plays a crucial role in the relationship among metabolism, immunity, and inflammation [7,83]. It is well established that concerning age there are alterations in both the composition and function of gut microbiota, which include reduced microbial diversity, depletion of SCFA-producing bacteria, and enrichment of potentially proinflammatory taxa [84,85]. Various human and animal literature has shown a reduced abundance of beneficial commensals like *Faecalibacterium prausnitzii*, *Akkermansia muciniphila*, *Lactobacillus*, and *Bifidobacterium* in the elderly, which are primary SCFA producers. This decrease is accompanied by an increase in pathobionts and pro-inflammatory bacteria like certain members of the phylum Bacteroidetes and Proteobacteria [85,86].

These mechanisms lead to a gradual deterioration of the integrity of the intestinal barrier, thereby promoting increased gut permeability and enhanced systemic exposure to microbial-derived products such as lipopolysaccharide (LPS) [17]. These mechanisms can be referred to as “metabolic endotoxemia,” which triggers the innate immune mechanism, specifically the TLR4/MyD88/NFκB signaling pathway, leading to the promotion of lower-grade persistent inflammation [87,88]. In the context of aging, this chronic inflammatory state is called inflammaging and is further exacerbated in individuals with T2DM, which accelerates the onset and severity of diabetic complications [89].

The combination of aging and diabetes creates a deleterious immunometabolic environment that is associated with immunosenescence, immune deregulation, and chronic inflammatory stimulation [90]. Immune dysfunction and adaptability combined with a dysfunctional gut microbiota contribute to endothelial activation, oxidative damage, and microvascular dysfunction [89]. These pathways are directly involved in the pathogenesis of severe complications from diabetes, such as nephropathy, neuropathy, retinopathy, and cardiovascular disease, which occur frequently and have a more rapid progression in older individuals with T2DM [91,92].

In addition, age-associated depletion of bioactive microbial metabolites, specifically SCFAs, deprives the host of their vital anti-inflammatory, barrier-stabilizing, and insulin-sensitizing functions [93,94,95]. Moreover, derangements in bile metabolism, coupled with the enhanced production of atherogenic metabolites such as trimethylamine oxide (TMAO), enhance systemic inflammation and cardiovascular risk among the elderly diabetic population [93].

Together, the combination of dysbiosis, the process of aging, and inflammaging offers a collective pathophysiological explanation for the predisposition of older T2DM patients to complications involving multiple organ systems [83]. The interaction between host/microbiome/immune system indicates that not only the use of microbiota-modifying therapies to improve glycemic outcomes but also the suppression of age-related inflammation and contributing immune dysfunction should be evaluated [96]. As illustrated in Figure 1, dysbiosis of the gut microbiota, characterized by decreased populations of bacteria that produce SCFAs and increased abundance of pathobionts, results in impaired intestinal barrier function and increased intestinal permeability. This facilitates metabolic endotoxemia through elevated circulating LPS levels, triggering chronic low-grade inflammation via activation of innate immune pathways, including TLR4–NF-κB signaling and oxidative stress. These processes converge to promote organ-specific microvascular and macrovascular complications affecting the kidney, retina, peripheral nerves, heart, and brain. Inflammaging and immunosenescence, both of which are aging phenomena, play a crucial role as amplifiers of these pathogenic pathways.

## 5. Discussion

This review highlights gut microbiota dysbiosis as a potential unifying pathophysiological contributor factor underlying the diverse spectrum of diabetic complications. Although individual complications have conventionally been studied separately, growing evidence suggests that common microbiota-driven mechanisms—including intestinal barrier integrity, endotoxin-mediated immune activation, chronic inflammation, oxidative stress, and dysregulated microbial metabolite production—contribute to both microvascular and macrovascular damage in T2DM [97,98]. However, most of the existing evidence comes from preclinical research and human observational research, but causality has yet to be proven.

Several common patterns of microbiota-derived changes have been observed across organ systems, including reduced abundance of SCFA-producing bacteria, increased levels of endotoxin-producing taxa (notably Gram-negative Enterobacteriaceae), and altered metabolism of bile acids and trimethylamine [99,100]. These changes culminate in a common inflammatory pathway, involving TLR signaling and NF-κB activation [17,101]. It appears that the interaction between gut microbiota and immunologic host function is a complex process; however, it is apparent that it represents an intricately self-fulfilling cycle with respect to the presence of metabolic disease [102,103].

Aging and inflammaging act as additional modifying variables in this framework, as older individuals with T2DM exhibit increased immune dysregulation, a decreased microbial population, and increased vulnerability to complications [104,105]. This convergent perspective emphasizes the need to shift from the glucose-centric view of diabetic complications towards a systems approach that takes into account host and microbiome interactions [83,106].

### 5.1. Clinical and Translational Implications

The identification of reproducible microbial signatures across diabetic complications emphasizes the utility of microbial profiling for both diagnostic and prognostic purposes [33]. Microbial metabolites like SCFAs, bile acids, and trimethylamine N-oxide can be used as biomarkers for disease severity and treatment response [107], together with microbiome signatures. In addition, interventions for gut dysbiosis such as dietary changes, prebiotics, probiotics, fecal microbiota transplantation, and microbiota-targeting medications have potential as supplements in the prevention and treatment of diabetes complications [4].

These findings further underpin the idea that the treatment of the gut microbiota by means of probiotics, prebiotics, fecal microbiota transplantation procedures, and immune-modulatory diets may not only provide metabolic control but also treat systemic inflammation and immune system dysfunction related to diabetic complications [108].

### 5.2. Future Directions

It is recommended that future efforts be directed to conduct longitudinal and mechanistic research to help define the causal relationship between specific microbial alterations and diabetic complication outcomes [76,109]. Large-scale prospective studies, combining multimicrobiome, metabolomics, transcriptomics, and immune system approaches, would be fundamental to the identification of reliable microbiome patterns as predictive biomarkers.

Nevertheless, the interpretation of gut microbiota results in patients with type 2 diabetes mellitus is complicated by the presence of various confounding factors, which might have a significant impact on the microbial profile and diversity. The use of common drugs such as metformin [7], antibiotics [4], statins [95], and proton pump inhibitors [110], along with diet [95], body mass index [95], impaired kidney function, age, gender [95], and other comorbid conditions, might play an important role in shaping the microbiome of individuals. Thus, these factors must be considered in future research to ensure the consistency and validity of results.

Randomized controlled trials should be designed to assess the efficacy of microbiota-targeted interventions, including personalized dietary strategy, prebiotics, probiotics, synbiotics, postbiotics, and fecal microbiota transplantation, in preventing or delaying the progression of diabetic complications [111,112]. In addition, age stratification of the population along with sex and the use of medications and other co-existing conditions can help in the development of precision medicine [113,114].

Lastly, the integration of microbiota profiling analysis into the conventional practice of medicine may provide the possibility of risk stratification and early intervention, especially among the vulnerable population of older patients with chronic T2DM [87].

### 5.3. Limitations of Current Evidence

It is important to note that, despite the presence of a substantial amount of evidence supporting the associations between dysbiosis, metabolic inflammation, and diabetic complications, direct evidence in humans between certain immune mechanisms (e.g., the TLR4/NF-κB pathway) and organ-specific endpoints is sparse and largely available in studies undertaken in animal models or ex vivo systems [87].

Similarly, though conditions driven by inflammation, such as IBD, do demonstrate the clinical importance of microbiota–immune interaction, further mechanistic and targeted studies are required to detail exactly how these mechanisms cross over into diabetic complications in diverse patient populations [108].

Limitations of this review include its narrative design and the heterogeneity of human microbiome studies. Also, human microbiome research can exhibit considerable heterogeneity across different populations studied, methods used, and analysis conducted. Thus, mechanisms described in this review are only biologically plausible and not causative.

Lastly, emerging evidence suggests that the gut virome and mycobiome are also altered in diabetes and its complications, also contributing to disease pathogenesis and progression [115,116,117,118]. Although these aspects were not addressed in the present review, they represent an important and relatively underexplored area that warrants further investigation and discussion in a separate review.

## 6. Conclusions

Gut microbiota dysbiosis is increasingly recognized as a potential pathological bridge connecting T2DM and its multiple systemic complications. Shared mechanisms, including, among others, disruption of intestinal barrier integrity, chronic low-grade inflammation, and imbalances in microbial metabolites, provide a common thread driving both micro- and macrovascular complications. Integrating microbiota-based diagnostics and therapeutics into precision medicine platforms, alongside conventional strategies, could provide new insights for optimal prevention and control of diabetic complications beyond glycemic control alone. In parallel, advances in artificial intelligence and high-throughput sequencing technologies are expected to accelerate progress in this field by enabling the identification and interpretation of complex microbiome signatures, ultimately facilitating more accurate risk stratification and personalized therapeutic interventions. More long-term studies are needed for validation and to determine the extent of the gut microbiota’s effect on diabetic complications.

## Figures and Tables

**Table 1:** Extended microbiota-linked complications of diabetes.

Complication	Mechanism	Microbial Signatures/Alterations
Nephropathy	SCFA reduction, endotoxemia	↑ Proteobacteria, *↓* Roseburia, ↑ systemic inflammation
Retinopathy	Gut–retina axis disruption	↑ *Alistipes*, ↓ antioxidant metabolites, microbial metabolites linked to oxidative stress
Neuropathy	Inflammation, SCFA dysregulation	↑ *Escherichia-Shigella*, ↓ *Faecalibacterium*, links to insulin resistance
Cerebrovascular Disease	Neuroinflammation via the gut–brain axis	TLR signaling, ↓ Clostridiales, ↑ Streptococcus; stroke associations
Cardiovascular Disease	TMAO production, gut permeability	↑ *Ruminococcus gnavus*, ↓ Lachnospiraceae; atherosclerosis risk
Peripheral Vascular Disease	Endotoxemia, LPS-induced inflammation	↑ *Lachnoclostridium*, ↓ Ruminococcaceae; promotes atherosclerosis
Reproductive System Disorders	Endothelial dysfunction via gut metabolites	↑ *Clostridium XVIII*, ↓ Alistipes; associated with erectile dysfunction

## Data Availability

No new data were created or analyzed in this study. Data sharing is not applicable to this article.
